# A Comparison of the Effects of Foam Rolling and Stretching on Physical Performance. A Systematic Review and Meta-Analysis

**DOI:** 10.3389/fphys.2021.720531

**Published:** 2021-09-30

**Authors:** Andreas Konrad, Markus Tilp, Masatoshi Nakamura

**Affiliations:** ^1^Institute of Human Movement Science, Sport and Health, Graz University, Graz, Austria; ^2^Institute for Human Movement and Medical Sciences, Niigata University of Health and Welfare, Niigata, Japan

**Keywords:** roller massage, strength, warm-up, myofascial release, stretching

## Abstract

Foam rolling and stretching with its various techniques are frequently used as a warm-up routine to increase the range of motion of a joint. While the magnitude of the changes in range of motion between foam rolling and stretching (static and dynamic techniques) is similar, it is not clear if this also holds true for performance parameters (e.g., strength, jump height). The purpose of this meta-analysis was to compare the effects of an acute bout of foam rolling (with and without vibration) with an acute bout of stretching (with all techniques included) on performance parameters in healthy participants. We assessed the results from 13 studies and 35 effect sizes by applying a random-effect meta-analysis. Moreover, by applying a mixed-effect model, we performed subgroup analyses with the stretching technique, type of foam rolling, tested muscle, treatment duration, and type of task. We found no significant overall effect, and the analysis revealed only a trend of the performance parameters in favor of foam rolling when compared to stretching (when considering all techniques). Significantly favorable effects of foam rolling on performance were detected with subgroup analyses when compared to static stretching, when applied to some muscles (e.g., quadriceps) or some tasks (e.g., strength), when applied for longer than 60 s, or when the foam rolling included vibration. When foam rolling was compared to dynamic stretching or applied in the non-vibration mode, the same magnitude of effect was observed. While the present meta-analysis revealed no significantly different effect between foam rolling and stretching (including all techniques) prior to exercise, differences could be observed under specific conditions.

## Introduction

The range of motion (ROM) of a joint can be acutely increased by both stretching exercises with various techniques (Konrad et al., 2017b, 2019; Konrad and Tilp, 2020b) and foam rolling exercises (Nakamura et al., 2021). Therefore, these two modalities are frequently used as warm-up routines in sports practice, especially in sports where a high ROM is needed (e.g., dance, martial arts). Studies which compared the effects on ROM between these modalities are inconclusive and reported either no difference between dynamic stretching and foam rolling (Somers et al., 2020), a favorable effect of foam rolling on ROM compared to static and dynamic stretching (Su et al., 2017), or a favorable effect of static stretching on ROM compared to foam rolling (Fairall et al., 2017). However, according to a recent meta-analysis (Wilke et al., 2020), the magnitude of the changes following stretching (including static and dynamic stretching) and foam rolling on ROM is the same. Thus, when the goal is to increase ROM, both stretching with its various techniques and foam rolling can be considered as adequate warm-up routines.

However, although a single application of stretching or foam rolling might also affect performance parameters, such as strength or jump height, to date, it is unclear as to which modality might have a favorable effect on performance. With regard to a single bout of stretching, it has been reported that, especially with static stretching techniques, longer stretching durations (e.g., ≥60 s) cause a more pronounced impairment in performance parameters (−4.6%) compared to stretching durations of <60 s (−1.1%) (Behm et al., 2016). With regard to other stretching techniques, Behm et al. (2016) reported a mean performance impairment of 3.7% immediately after proprioceptive neuromuscular facilitation (PNF) stretching, but an increase in performance of 1.3% after dynamic stretching. Thus, it can be concluded that the effects of a single stretching exercise on performance are highly dependent on the stretch duration and stretching technique (Behm and Chaouachi, 2011; Kay and Blazevich, 2012; Behm et al., 2016), but are likely also dependent on the muscles stretched [e.g., see the review of the influence of hip flexor muscles in Konrad et al. (2021b)]. A possible mechanism for a detrimental effect in performance can be found in a decrease in muscle stiffness, especially following longer durations of static stretching (Kay et al., 2015; Konrad et al., 2017a,b). Hence, this decrease in muscle stiffness might negatively affected force production (Trajano et al., 2019; Monte and Zignoli, 2021). Although detrimental effects have been reported for static and PNF stretching [e.g., Behm et al. (2016)], with regard to the acute effects of foam rolling on performance, a recent meta-analysis (Wiewelhove et al., 2019) reported no such detrimental effects. In their meta-analysis, Wiewelhove et al. (2019) even reported a tendency of improvement (*P* = 0.06) in sprint performance (+0.7%), but negligible effects in jump or strength performance. This is in accordance with another review by Cheatham et al. (2015), who reported that a single bout of a foam rolling exercise likely does not induce changes in performance parameters.

A recent meta-analysis revealed that changes in ROM have the same magnitudes when comparing a single stretching exercise (including static and dynamic stretching) or a single foam rolling exercise (Wilke et al., 2020). However, to date a meta-analysis which compares the effects of stretching and foam rolling on performance parameters is still missing. A conduction of such a meta-analysis would allow to establish the big picture about the different effects of these warm-up modalities. Thus, the purpose of this meta-analysis was to compare the effects of an acute bout of foam rolling with an acute bout of various stretching techniques on performance parameters (e.g., strength, jump height) in healthy participants.

## Materials and Methods

This review was conducted according to the PRISMA guidelines and the suggestions from Moher et al. (2009) for systematic reviews with meta-analysis.

### Search Strategy

**Figure 1:**
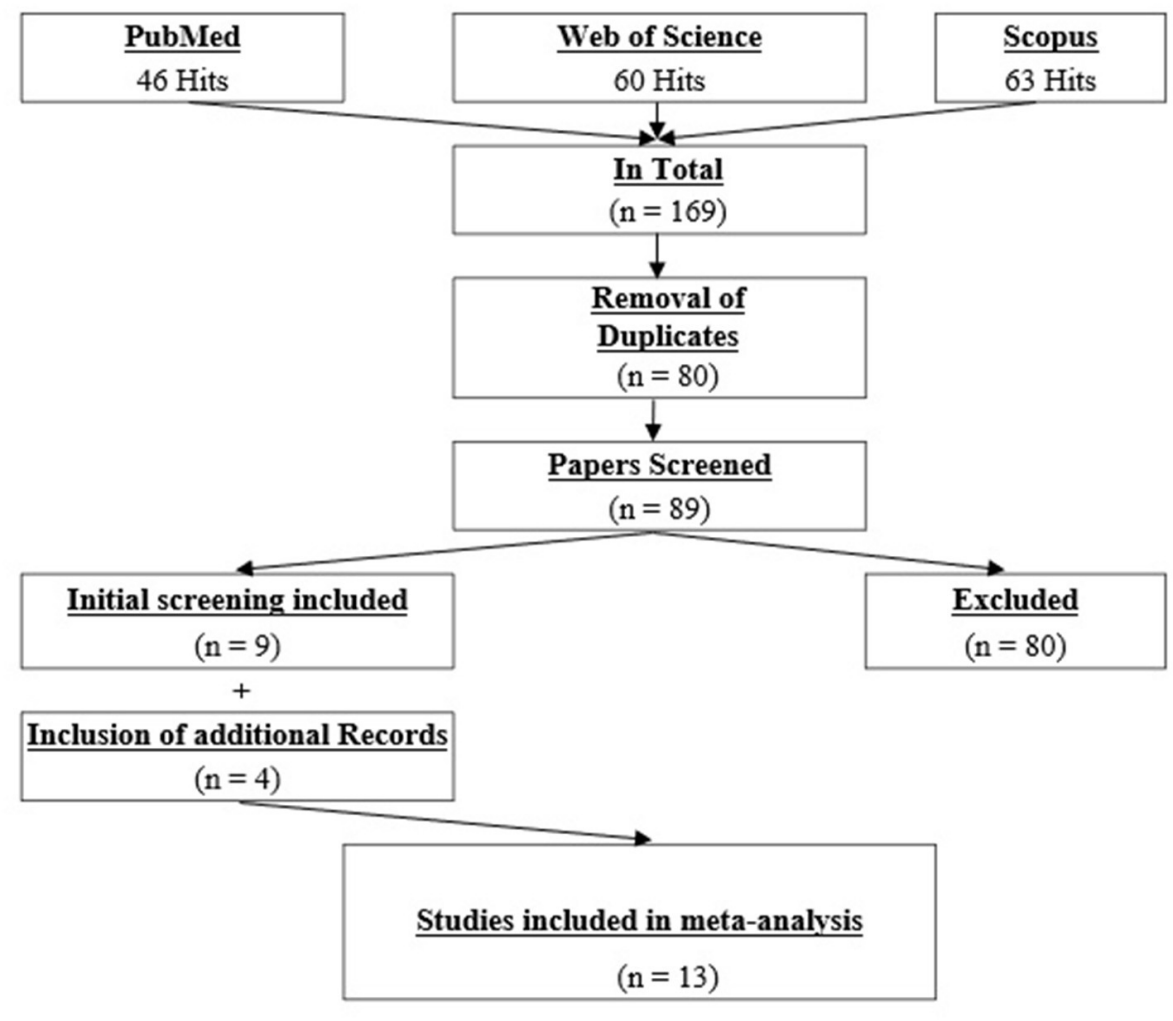
PRISMA flowchart.

An electronic literature search was performed in PubMed, Scopus, and Web of Science. The search period ranged from 1990 until 15th February 2021. The keywords for the online search were (“foam rolling” OR “self-myofascial release” OR “roller massage” OR “foam roller”) AND (stretch^*^), and were the same for all the databases. The systematic search was done by two independent researchers (AK, MN). In the first step, all the hits were screened by their abstract. If the content of a study remained unclear, the full text was screened to identify the relevant papers. Following this independent screening process, the researchers compared their findings. Disagreements were resolved by jointly reassessing the studies against the eligibility criteria. Overall, 169 papers were screened, from which nine papers were found to be eligible for this review. However, following the additional search of the references (search through the reference list) and citations (search through Google Scholar) of the nine already included papers, four more papers were identified as relevant. Therefore, in total, 13 papers were included in this systematic review. The whole search process is illustrated in Figure 1.

### Inclusion and Exclusion Criteria

This review considered studies that investigated the effects of an acute bout of both stretching and foam rolling on performance parameters (e.g., strength, jump height) in healthy participants. We included studies in English, German, and Japanese language with crossover (pre to post comparison or post comparison) or parallel group (pre to post comparison) designs. However, we excluded studies which investigated the combined effects of stretching and foam rolling, and studies of other myofascial techniques than foam rolling or roller massage, and conference papers or theses.

### Extraction of the Data

From the included papers, the characteristics of the participants, the sample size, the study design, the characteristics of the intervention (e.g., stretching technique, vibration foam rolling vs. non-vibration foam rolling, duration) and the results of the main variables (performance parameters) were extracted. For the performance parameters, either the pre and post values (plus standard deviations) or the post values (plus standard deviations) of both the stretching and the foam rolling group were extracted. If the required data were missing in the included studies, the authors of the studies were contacted *via* email.

### Statistics and Data Synthesis

The meta-analysis was performed using Comprehensive Meta-Analysis software, according to the recommendations of Borenstein et al. (2009). By the use of a random-effect meta-analysis, we assessed the effect size in terms of the standardized mean difference. Moreover, by using a mixed-effect model, we performed subgroup analyses with the stretching technique (static stretching, dynamic stretching), the type of foam rolling (vibration foam rolling, non-vibration foam rolling), the tested muscle (adductor, hamstrings, quadriceps, triceps surae, whole-body movement), the treatment duration (>60 s, ≤ 60 s), the type of task (strength, jump height, sprinting, endurance), and the activity level of the participants (physical active vs. well-trained/professional). To determine if there were differences between the effect sizes of the subgroups, Q-statistics were applied (Borenstein et al., 2009). According to the recommendations of Hopkins et al. (2009), we defined the effects for a standardized mean difference of <0.2, 0.2–0.6, 0.6–1.2, 1.2–2.0, 2.0–4.0, and >4.0 as trivial, small, moderate, large, very large, and extremely large, respectively. *I*^2^ statistics were calculated to assess the heterogeneity among the included studies, and thresholds of 25, 50, and 75% were defined as having a low, moderate, and high level of heterogeneity, respectively (Higgins et al., 2003; Behm et al., 2021). An alpha level of 0.05 was defined for the statistical significance of all the tests.

### Risk of Bias Assessment and Methodological Quality

The methodological quality of the included studies was assessed using the PEDro scale. In total, 11 methodological issues were assessed by the two independent researchers (AK, MN) and assigned with either one or no point. Hence, studies with a higher score represent a higher methodological quality. If any conflict between the ratings of the two researchers was found, the methodological issues were reassessed and discussed. Moreover, statistics of the Egger's regression intercept test and visual inspection of the funnel plot were applied to detect possible publication bias.

## Results

### Results of the Search

In total, 13 studies investigated the effects of both a single foam rolling exercise and a single stretching exercise on performance parameters, and hence were included in the meta-analysis. Overall, 35 effect sizes could be extracted from these studies. In summary, 304 participants (202 males and 102 females) with a mean age of 21.3 (±1.8 years) participated in the included studies. Table 1 presents the characteristics and outcomes of the 13 studies.

**Table 1:** Characteristics of the included studies (*n* = 13).

**Study**	**Participants**	**Type of stretching**	**Type of foam rolling**	**Application per muscle group (sec)**	**Outcome**
Behara and Jacobson (2017)	*N* = 14; 14 male well-trained NCAA Division 1 football offensive linemen (age 20.04 ± 1.41 years)	Dynamic	Non-vibration	60	Peak vertical jump power (W)
					Average vertical jump power (W)
					Average vertical jump velocity (ms^∧^−1)
					Peak vertical jump velocity (ms^∧^−1)
					Peak isometric leg extension torque (Nm)
					Average isometric leg extension torque (Nm)
					Average isometric leg flexion torque (Nm)
					Peak isometric leg flexion torque (Nm)
Connolly et al. (2020)	*N* = 40; 20 male (age 22.5 ± 1.8 years) and 20 female (age 23.6 ± 4.2 years) physically active	Static	Non-vibration	60	Maximum voluntary contraction torque (Nm)
Folli et al. (2021)	*N* = 29; 23 male and 6 female athletes (age 16 ± 1.14 years)	Static	Non-vibration	60	Maximum voluntary contraction torque (Nm)
Halperin et al. (2014)	*N* = 14; 12 male (age 23 ± 4 years) and 2 female (age 22 ± 3 years) physically active	Static	Non-vibration	90	Maximum voluntary contraction force (*N*)
Janot et al. (2013)	*N* = 23; 9 males and 14 females (age 20.3 ± 1.4 years); activity level not reported	Static	Non-vibration	90	Peak power output (W)
					Relative peak power output (W/kg)
					Average power output (W)
					Relative average power output (W/kg)
					Minimum power output (W)
					Relative minimum power output (W/kg)
					Percentage power drop (%)
Kopec et al. (2017)	*N* = 20; 10 male and 10 female physically active (age 22.5 ± 4 years)	Dynamic	Non-vibration	30	Vertical jump height (cm)
Lee et al. (2018)	*N* = 30; 30 male college students (age 20.4 ± 1.2 years)	Static	Vibration, non-vibration	90	Relative quadriceps strength (Nm/Kg)
					Relative hamstring strength (Nm/Kg)
Lopez-Samanes et al. (2021)	*N* = 11; 11 male professional tennis players (age 20.6 ± 3.5 years)	Dynamic	Non-vibration	60	Counter movement jump (cm)
					10 m sprint (s)
					Agility (s)
Lyu et al. (2020)	*N* = 20; 20 male recreationally active (age 21 ± 1.01 years)	Static	Vibration	90	Relative plantar flexor strength (Nm/kg)
					Agility (s)
Pişirici et al. (2020)	*N* = 28; each group (stretching, foam rolling) 14 (7 male and 7 female) recreationally active with an average age of 21.5 ± 1.6 years, 22.7 ± 3.8 years, respectively	Dynamic	Non-vibration	180	Vertical jump height (cm)
Sagiroglu et al. (2017)	*N* = 16; 16 male well-trained combat athletes (age 23.9 ± 3.7 years)	Static	Non-vibration	60	Counter movement jump (cm)
Smith et al. (2018)	*N* = 29; 8 male and 21 female (23 physically active/6 sedentary) (age 22 ± 3 years)	Dynamic	Non-vibration	90	Vertical jump height (cm)
Su et al. (2017)	*N* = 30; 15 male and 15 female college students (age 21.43 ± 1.48 years)	Dynamic, static	Non-vibration	90	Relative quadriceps strength (Nm/Kg)
					Relative hamstring strength (Nm/Kg)

### Risk of Bias Assessment and Methodological Quality

**Figure 2:**
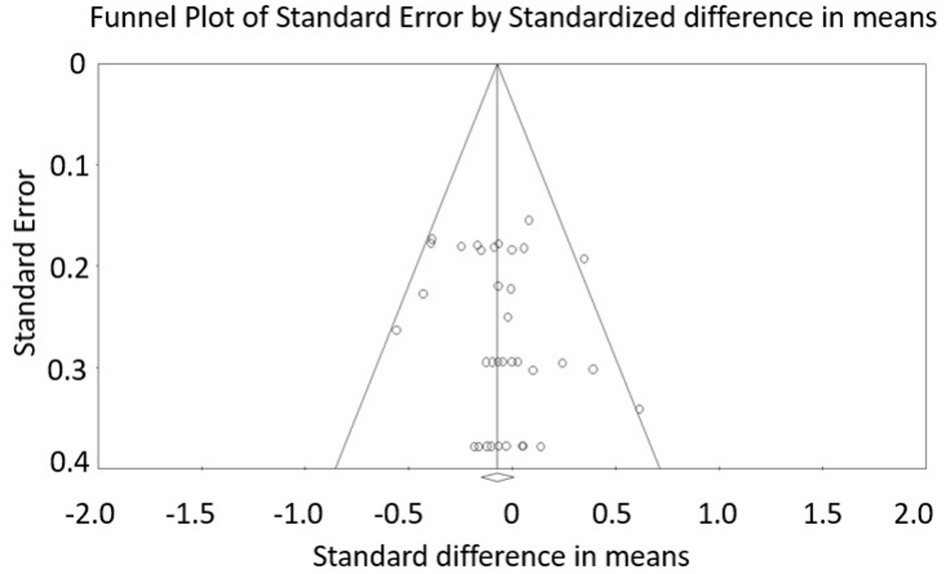
Funnel plot analysis.

Figure 2 shows the funnel plot, including all 35 effect sizes included in this meta-analysis. A visual inspection and the Egger's regression intercept test (intercept 0.68; *P* = 0.21) indicate that no reporting bias is likely. The methodological quality, as assessed with the PEDro scale, reveals a range of scores between 7 and 8 points (out of 10) for all the included studies. The average PEDro score value is 7.15 (±0.38), indicating a low risk of bias (Maher et al., 2003; Moran et al., 2021). The two assessors agreed with 96.5% out of the 143 criteria (13 studies × 11 scores). The mismatched outcomes were discussed and the assessors finally agreed on the scores presented in Table 2.

**Table 2:** PEDro scale of the included studies.

**Study**	Inclusion criteria^*^	**Random allocation**	**Concealed allocation**	**Similarity at baseline**	**Subject blinding**	**Therapist blinding**	**Assessor blinding**	**>85% follow-up**	**Intention to treat analysis**	**Between-group comparison**	**Point estimates and variability**	**Total**
Behara and Jacobson (2017)	1	1	0	1	1	0	0	1	1	1	1	7
Janot et al. (2013)	1	1	0	1	1	0	1	1	1	1	1	8
Su et al. (2017)	1	1	0	1	1	0	0	1	1	1	1	7
Smith et al. (2018)	1	1	0	1	1	0	0	1	1	1	1	7
Folli et al. (2021)	1	1	0	1	1	0	0	1	1	1	1	7
Kopec et al. (2017)	1	1	0	1	1	0	0	1	1	1	1	7
Connolly et al. (2020)	1	1	0	1	1	0	0	1	1	1	1	7
Pişirici et al. (2020)	1	1	0	1	1	0	1	1	1	1	1	8
Lyu et al. (2020)	1	1	0	1	1	0	0	1	1	1	1	7
Lee et al. (2018)	1	1	0	1	1	0	0	1	1	1	1	7
Lopez-Samanes et al. (2021)	1	1	0	1	1	0	0	1	1	1	1	7
Halperin et al. (2014)	1	1	0	1	1	0	0	1	1	1	1	7
Sagiroglu et al. (2017)	1	1	0	1	1	0	0	1	1	1	1	7

* *Was not counted for the final score; 1, one point awarded; 0, no point awarded*.

### Overall Effects

**Figure 3:**
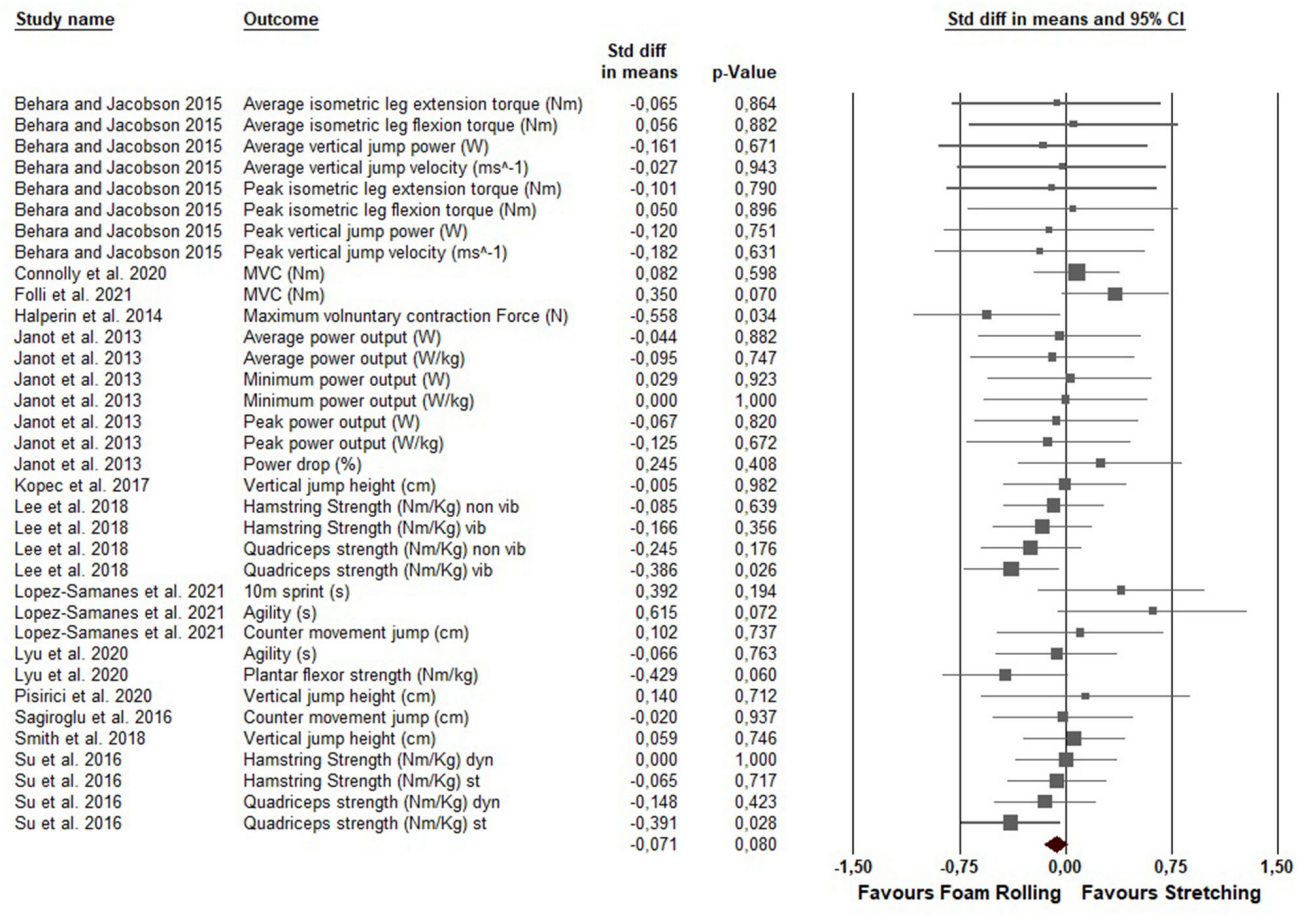
Forest plot presenting 35 effect sizes. Std diff in means, standardized difference in means; CI, confidence interval.

The average percentage change in performance (pre to post or post to control) in the included studies following a single foam rolling treatment was an increase of 2.19% [CI (95%) 0.15–4.38%]. In addition, a single stretching exercise led to an average increase of 1.11% [CI (95%) −0.80–3.09%] in performance parameters in the included studies. The meta-analysis revealed a non-significant difference between the two modalities [ES = −0.071; *Z* = −1.748; CI (95%) −0.150–0.009; *P* = 0.08; *I*^2^ = 0.0]. Figure 3 presents the forest plot of the meta-analysis, listed in alphabetical order of the author names.

### Subgroup Analysis

A summary of all the subgroup analyses is provided in Table 3.

**Table 3:** Statistics of the subgroup analysis.

**Subgroup**	**Number of measures**	**Std diff in means (95% CI)**		***P***	***Q* statistics**
**Stretching technique (vs. foam rolling)**					
Dynamic stretching	16	0.025	(−0.112 to 0.163)	0.717	
Static stretching	19	−0.118	(−0.221 to −0.015)	0.024^*^	
Overall	*35*	–*0.067*	*(*–*0.149 to 0.016)*	*0.112*	(*Q* = 2.686; *df* (Q) = 1; *P* = 0.101)
**Rolling technique (vs. stretching)**					
Non-vibration	31	−0.031	(−0.118 to 0.056)	0.489	
Vibration	4	−0.265	(−0.457 to −0.073)	0.007^*^	
*Overall*	*35*	–*0.071*	*(*–*0.150 to 0.009)*	*0.08*	(*Q* = 4.738; *df* (*Q*) = 1; *P* = 0.03)^#^
**Muscle tested (foam rolling vs. stretching)**					
Adductors	1	0.082	(−0.222 to 0.385)	0.598	
Hamstrings	7	0.002	(−0.151 to 0.156)	0.977	
Quadriceps	6	−0.275	(−0.441 to −0.109)	0.001^*^	
Triceps Surae	2	−0.484	(−0.821 to −0.146)	0.005^*^	
Whole body movement	19	0.032	(−0.096 to 0.161)	0.624	
*Overall*	*35*	–*0.071*	*(*–*0.150 to 0.009)*	*0.08*	(*Q* = 15.856; *df* (*Q*) = 4; *P* = 0.003)^#^
**Treatment duration (foam rolling vs. stretching)**					
>60 s	20	−0.149	(−0.245 to −0.053)	0.002^*^	
≤ 60 s	15	0.101	(−0.041 to 0.243)	0.164	
*Overall*	*35*	–*0.071*	*(*–*0.150 to 0.009)*	*0.08*	(Q = 8.164; df (Q) = 1; P = 0.004)#
**Tasks (foam rolling vs. stretching)**					
Strength	16	−0.136	(−0.250 to −0.022)	0.019^*^	
Jump	9	0.000	(−0.185 to 0.185)	0.998	
Speed	3	0.252	(−0.162 to 0.665)	0.234	
Endurance	7	−0.008	(−0.227 to 0.210)	0.94	
*Overall*	*35*	–*0.069*	*(−0.156 to 0.017)*	*0.117*	(*Q* = 4.447; *df* (*Q*) = 3; *P* = 0.214)
**Activity level (physical active vs. well trained/professional)**					
Physical active	12	−0.076	(−0.214 to 0.063)	0.283	
Well trained/professional	12	0.064	(−0.130 to 0.258)	0.519	
*Overall*	*24*	–*0.029*	*(*–*0.499 to 0.618)*	*0.618*	(*Q* = 1.322; *df* (*Q*) = 1; *P* = 0.250)

*Negative values of Std diff (= standardized difference) in means indicates a favorable effect for foam rolling (and vice versa).*

# *Significant difference between groups.*

* *Significant difference within a group*.

### Stretching Technique

The subgroup analysis of the stretching technique (static stretching, dynamic stretching) revealed no significant difference in performance parameters by comparing the subgroups of “dynamic stretching vs. foam rolling” and “static stretching vs. foam rolling” (*P* = 0.101; *Q* = 2.686). However, the subgroup of “static stretching vs. foam rolling” showed a significant effect on performance parameters in favor of foam rolling compared to static stretching [ES = −0.118; CI (95%) −0.221 to −0.015; *P* = 0.02].

### Vibration vs. Non-vibration Foam Rolling

By comparing the different rolling techniques (vibration, non-vibration), the subgroup analysis revealed a significant difference between the subgroups of “non-vibration foam rolling vs. stretching” and “vibration foam rolling vs. stretching” (*P* = 0.03; *Q* = 4.738). While non-vibration foam rolling showed the same magnitude of change as stretching, the vibration foam rolling exercise revealed a significantly better effect than stretching [ES = −0.265; CI (95%) −0.457 to −0.073; *P* = 0.007].

### Muscle-Specific Analysis

A further subgroup analysis tested for differences in the muscles involved in the performance tests (adductors, hamstrings, quadriceps, triceps surae, whole-body movement). There was a significant difference between the muscles tested when comparing stretching with foam rolling (*P* = 0.003; *Q* = 15.856). Meanwhile, there were no differences in the magnitude of change for the adductors, hamstrings, and whole-body movement when comparing stretching and foam rolling. However, the quadriceps and triceps surae revealed a significant favorable effect for foam rolling when compared to stretching [ES = −0.275; CI (95%) −0.441 to −0.109; *P* = 0.001] and [ES = −0.484; CI (95%) −0.821 to −0.146; *P* = 0.005]).

### Treatment Duration (>60 vs. ≤ 60 s)

The subgroup analysis for the duration of both foam rolling and stretching (>60 s, ≤ 60 s) revealed a significant difference (*P* = 0.004; *Q* = 8.164). When applied for ≤ 60 s, foam rolling and stretching showed the same magnitude of change. However, durations of more than 60 s resulted in a significantly better effect for foam rolling than stretching [ES = −0.149; CI (95%) −0.245 to −0.053; *P* = 0.002].

### Task-Specific Analysis

The task-specific (strength, jump height, speed, endurance) subgroup analysis revealed no significant difference between the tasks when comparing the acute effects of stretching with the acute effects of foam rolling on performance parameters (*P* = 0.214; *Q* = 4.447). Although, no significant difference between tasks was found, the strength task showed a significant favorable effect for foam rolling when compared to stretching [ES = −0.136; CI (95%) −0.250 to −0.022; *P* = 0.019].

### Activity Level

The subgroup analysis of the activity level revealed no significant difference between the subgroups (physical active vs. well-trained/professional) when comparing the acute effects of stretching with the acute effects of foam rolling on performance parameters (*P* = 0.250; *Q* = 1.322). Moreover, the same magnitudes were found following stretching and foam rolling on performance parameters within the activity level groups.

## Discussion

The purpose of this review was to compare the effects of an acute bout of stretching or foam rolling on performance parameters (e.g., maximum voluntary contractions, jumping performance, sprinting performance) in healthy subjects. The meta-analysis revealed no significant differences in the overall effects on performance between stretching and foam rolling (ES = −0.071, *P* = 0.08; see also Figure 3). However, a subgroup analysis showed a significant greater effect of foam rolling compared to static stretching on performance parameters (ES = −0.118; *P* = 0.02), while dynamic stretching showed the same magnitude of change as foam rolling (ES = 0.025; *P* = 0.71). Furthermore, the subgroup analysis revealed a significant greater effect for vibration foam rolling compared to stretching (ES = −0.265; *P* = 0.007), but the same magnitude of change when non-vibration foam rolling was compared with stretching (ES = −0.031; *P* = 0.49). Moreover, when the treatment was applied to the quadriceps or triceps surae muscles, foam rolling showed a significant greater effect on performance when compared to stretching (ES = −0.275; *P* = 0.001 and ES = −0.484; *P* = 0.005). At higher durations of application (>60 s), foam rolling showed a significant greater effect when compared to stretching (ES = −0.149; *P* = 0.002). By distinguishing between the performance tasks, only the strength tasks showed a significant greater effect following foam rolling when compared to stretching (ES = −0.136; *P* = 0.02). Concerning the activity levels, no differences in the effects between and within the levels were observed (*P* = 0.250; *Q* = 1.322).

Meta-analyses of the acute effects of stretching have previously reported detrimental effects on performance, especially when applied with the static stretching technique and for a long duration (>60 s) (Behm et al., 2016). However, by summarizing the results for dynamic stretching, 1.3% increases in performance have also been reported (Behm et al., 2016). With regard to the evidence on the acute effects of single foam rolling treatments, a recent meta-analysis reported that performance did not change or even tended to increase (Wiewelhove et al., 2019). Hence, we assume that foam rolling may have a superior effect on performance when compared to stretching. However, the pooled effect size was not significantly different and showed only a trivial tendency for a favorable effect for foam rolling when compared to stretching (ES = −0.071; *P* = 0.08). Specifically, our analysis does not provide clear evidence that foam rolling increases performance, as reported in a different meta-analysis (Wiewelhove et al., 2019). Pre to post comparisons (or comparisons to the controls) of the 13 included studies revealed an average increase in performance parameters of 2.19% [CI (95%) 0.15–4.38%] following the foam rolling treatment, and an average increase following stretching of 1.11% [CI (95%) −0.80 – 3.09%]. Hence, our results tend to show that foam rolling has more potential to increase performance than stretching. However, a possible explanation for the lack of a significant difference between the two modalities is likely based on the fact that the included studies varied substantially in their interventions. These variations were e.g., the stretching technique (static stretching, dynamic stretching), the foam rolling technique (with and without vibration), or the treatment duration (30–180 s). Since the superior effect of dynamic stretching compared to static stretching on performance has been reported (Behm and Chaouachi, 2011), a goal of one of the subgroup analyses was to distinguish between foam rolling and these two stretching modalities. This revealed the same magnitude of change in performance for dynamic stretching {average change: +1.51% [CI (95%) −1.55 to 5.42%]} when compared to foam rolling {+1.71%; [CI (95%) −1.17 to 5.14%]}; however, static stretching showed only a statistically significant trivial detrimental effect (ES = −0.12; *P* = 0.02) when compared to foam rolling. The pre to post comparison (or post to control comparison) of the included studies on static stretching revealed an average increase of +0.60% [CI (95%) −0.85 to 2.09%], while the corresponding average change for foam rolling was an increase of +2.74% [CI (95%) 0.42–5.11%]. This result indicates that either dynamic stretching or foam rolling should be applied as a warm-up routine rather than static stretching if the goal is to optimize performance. A possible mechanism for the favor effect of dynamic stretching and foam rolling compared to static stretching might be the different mechanical effects of the different methods on the muscle-tendon unit. When applied with a sufficient amount of time [e.g., >60 s (Konrad et al., 2017b; Konrad and Tilp, 2020a)], there is evidence that a single static stretching exercise can lead to a decrease in soft tissue compliance. While some authors reported a decrease in muscle stiffness following a single static stretch (Kay et al., 2015; Konrad et al., 2017a,b), others reported a decrease in tendon stiffness (Kubo et al., 2001; Kato et al., 2010). However, studies of dynamic stretching did not report such changes in the compliance of the muscle-tendon unit (Mizuno and Umemura, 2016; Kaneda et al., 2020), which likely indicated changes in stretch tolerance as a mechanism for the changes in ROM. While some studies reported a decrease in muscle stiffness following a single foam rolling exercise (Morales-Artacho et al., 2017), others reported no changes in muscle stiffness (Mayer et al., 2019). Since muscle stiffness is positively related to the rate of force development (Monte and Zignoli, 2021), a decreased muscle stiffness can negatively affect force production, which has been frequently reported following long-duration static stretching in recent years (e.g., Konrad et al., 2019).

However, it should be noted that the results of the current meta-analysis are based on stretching or foam rolling interventions without any further warm-up. While the main mechanism for an increase in ROM following both stretching and foam rolling seems to be an increased stretch tolerance, Wiewelhove et al. (2019) hypothesized that the performance differences between foam rolling and static stretching are probably due to the additional warm-up or placebo effect of foam rolling. When a static stretching exercise of up to 120 s is followed by a sport-specific post stretching activation, no detrimental effect on performance has been reported by various studies (Samson et al., 2012; Behm et al., 2016; Reid et al., 2018). Reid et al. (2018) showed such a favorable effect in performance parameters (e.g., strength or jump height) in several static stretching conditions (30, 60, 120 s, no stretching) when combined with a sport-specific post stretching activation (30 s each of gluteal kicks and high knees, and 60 s each of walking hip openers, dynamic leg kicks to opposing hand, walking lunges with rotation, and the inchworm exercise), compared to the same stretching conditions without sport-specific post stretching activation. Moreover, Samson et al. (2012) reported an increase in sprint speed after the combination of stretching (static or dynamic) and a sport-specific post stretching activation (high knee skippings, high knee running, and butt kick running; done twice for 20 m). However, no change was observed when the stretching was combined with a general warm-up (5 min of running on a 200 m track at 70% of the individual's age-predicted heart rate). With regards to flexibility, static stretching was found to be more efficient in the sit and reach test than dynamic stretching, when both were combined with sport-specific post stretching activation. Hence, in sports where both strength and flexibility are needed, static stretching combined with sport-specific post stretching activities might be the proper solution for a warm-up routine. To the best of the authors' knowledge, to date, the efficacy of such sport-specific post activation has not been tested following a single foam rolling exercise. This additional application of a specific warm-up routine immediately following foam rolling might have a similar or even a favorable effect on performance when compared to stretching. Hence, we recommend that future studies should take this into consideration.

Concerning vibration and non-vibration foam rolling, Wilke et al. (2020) speculated in their meta-analysis that vibration foam rolling might have a favorable effect compared to non-vibration foam rolling when the goal is to increase the ROM of a joint. Our present meta-analysis showed a small significant effect in favor of vibration foam rolling on performance when compared to stretching (ES = −0.27; *P* = 0.007). The percentage changes following the vibration foam rolling revealed an average increase of +6.37% [CI (95%) −2.85 to 9.89%], while stretching showed an average change of −0.58% [CI (95%) −1.48 to 0.32%]. In contrast, the magnitude of the change in performance was the same in non-vibration foam rolling {+1.67% [CI (95%) −0.32 to 3.80%]} and stretching {+1.27% [CI (95%) −0.56 to 3.40%]}. To date, several reports have underlined these findings and reported either a superior effect of vibration foam rolling on performance parameters compared to non-vibration foam rolling (Lee et al., 2018; Reiner et al., 2021) or stretching (Lee et al., 2018; Lyu et al., 2020). A possible mechanism could be that vibration therapy can stimulate more muscle receptors in the three afferent fiber types (Ia, II, and Ib), which leads to an increase in motor fiber recruitment (Fallon and Macefield, 2007; Germann et al., 2018; Reiner et al., 2021). Moreover, it has been suggested that the possibly superior effect of vibration foam rolling compared to non-vibration foam rolling on performance parameters could be due to the greater contribution of the mechanoreceptors at higher vibration frequencies (Behm and Wilke, 2019; Reiner et al., 2021). However, caution has to be taken since our findings are based on only four effect sizes in vibration foam rolling. Hence, there is a need to conduct further studies of the effect of vibration foam rolling compared to other modalities (non-vibration foam rolling or stretching) on performance parameters.

When a performance test (and the treatment) was applied to the quadriceps or triceps surae muscle, foam rolling showed a small effect on performance when compared to stretching (quadriceps: ES = −0.275; *P* = 0.001; triceps surae: ES = −0.484; *P* = 0.005). However, this was not the case when the tests included whole-body movements (e.g., jumps) or primarily included the activity of the hamstring or adductor muscles. It must be noted that the results of this subgroup analysis are based on only a few effect sizes (adductor muscle: 1, triceps surae muscle: 2). Thus, caution should be taken not to overemphasize these results. For the whole-body movements (*n* = 19 effect sizes), an increase was observed following both stretching {+2.41% [CI (95%) 0.37–5.14%]} and foam rolling {+2.47% [CI (95%) 0.51–5.17%]}. The performance of the hamstring muscle (*n* = 7) decreased by −0.61% [CI (95%) −4.40–3.36%] and −2.42% [CI (95%) −5.98–1.23%] following stretching and foam rolling, respectively. When the quadriceps muscles were tested (*n* = 6), there was an average increase of +3.00% [CI (95%) −1.35–7.36%] following foam rolling but a decrease of −1.21% [CI (95%) −4.28–1.89%] following stretching. The effect sizes for the hamstring and quadriceps performance tests were mainly based on three studies where stretching and foam rolling were performed on both the quadriceps and the hamstrings (Behara and Jacobson, 2017; Su et al., 2017; Lee et al., 2018). Such different muscle-specific changes (quadriceps vs. hamstrings) in performance are difficult to explain and should be addressed in future studies.

With regard to the duration of the application, our meta-analysis showed that intervention durations of >60 s showed a trivial significant greater effect for foam rolling on performance compared with stretching (ES = −0.149; *P* = 0.002). However, this was not the case for an application duration of ≤ 60 s. According to several reviews, stretching for ≤ 60 s likely has no detrimental effects on performance parameters (Kay and Blazevich, 2012; Behm et al., 2016; Konrad et al., 2021b), while stretching for more than 60 s likely has a detrimental effect (Kay and Blazevich, 2012; Behm et al., 2016) due to a decrease in muscle stiffness (Kay et al., 2015; Konrad et al., 2017a,b) and hence, a negatively affected force production (Monte and Zignoli, 2021). For this reason, one could have assumed that, in the included studies of the present analysis with stretching durations of >60 s, the performance would decrease, while this would not be expected following foam rolling. However, although the meta-analysis revealed a favorable effect of foam rolling compared to stretching following more than 60 s of the application, we observed an average increase of performance in both stretching {+1.88% [CI (95%) −0.16 to 5.25%]} and foam rolling {+4.79% [CI (95%) 2.40 to 7.73%]}. Intervention durations of ≤ 60 s caused only a trivial change in performance in both groups {stretching: 0.04% [CI (95%) −2.32 to 2.24%]; foam rolling: −0.82% (CI (95%) −3.09 to 1.33%]}. Thus, likely due to an additional warm-up effect suggested by Wiewelhove et al. (2019), it can be assumed that a longer duration of foam rolling might lead to an increase in performance, indicating a dose-response effect. With regard to the ROM, Bradbury-Squires et al. (2015) reported a trend (*P* = 0.08) of longer foam rolling durations leading to greater increases in ROM compared to shorter durations of foam rolling. In sports where both a high ROM and strength are needed to achieve high performance, foam rolling for longer durations (>60 s) might be an appropriate warm-up tool, while longer stretching durations should be avoided.

With regard to the various tasks included in this meta-analysis, a favorable trivial effect for foam rolling {+1.92% [CI (95%) −1.52 to 5.43%]} compared to stretching {−0.81% [CI (95%) −3.04 to 1.47%]} was found in the strength tasks, but not in the jump height, speed, or endurance tasks. A reason for this could be the involvement of muscles in different tasks. In contrast to strength tasks, where the tests considered primarily single muscle or joint performance, jump height, speed, and endurance tasks involve the use of various muscles, which are likely not all treated during the stretching or foam rolling intervention. Therefore, it can be assumed that muscle stretching applied with a long duration [>60 s (Behm et al., 2016)] will rather lead to a reduction in strength of the stretched muscle than to a reduction in a more complex movement including several muscles. If stretching, and also foam rolling, could induce changes in strength measures of specific muscles, this might not affect jump height (Konrad et al., 2020) or endurance performance (Giovanelli et al., 2018; Konrad et al., 2021a) significantly.

The subgroup analysis of the activity level revealed no significant difference between (*P* = 0.250; *Q* = 1.322) and within the activity levels (physical active vs. well-trained/professional) when comparing the acute effects of stretching with foam rolling on performance parameters. This was a surprising finding since previous studies reported (Arampatzis et al., 2007; Konrad and Tilp, 2018) that well-trained athletes had a different muscle architecture and/or strength parameters compared to their non-active peers. Moreover, Donti et al. (2019) found that following a 60 s static stretching intervention of two different athletes' groups (rhythmic gymnasts and volleyball players) the ROM increased. However, the ROM changes in rhythmic gymnasts were even higher. This was associated with a greater fascicle elongation and a greater muscle tendon junction displacement in the rhythmic gymnasts (Donti et al., 2019). Hence, future studies should investigate the differences between stretching and foam rolling on ROM and performance parameters in different populations (e.g., professional athletes vs. physical active or sedentary people).

## Conclusion

In conclusion, while the present meta-analysis revealed no significantly different effect between foam rolling and stretching prior to exercise, differences could be observed under specific conditions. In particular, favorable effects of foam rolling on performance have been detected, when compared to static stretching, when applied to some muscles (e.g., quadriceps), or some tasks (e.g., strength), when applied for longer than 60 s, or if the foam rolling included vibration. When foam rolling was compared to dynamic stretching or applied in the non-vibration mode, the same magnitude of change was observed.

## Data Availability Statement

The original contributions presented in the study are included in the article/supplementary material, further inquiries can be directed to the corresponding authors.

## Author Contributions

AK and MN collaborated on the literature review and in producing the figures and tables. AK performed the meta-analysis. All authors collaborated on interpreting the results and writing the manuscript and contributed to the article and approved the submitted version.

## Funding

This study was supported by a grant (Project J 4484) from the Austrian Science Fund FWF.

## Conflict of Interest

The authors declare that the research was conducted in the absence of any commercial or financial relationships that could be construed as a potential conflict of interest.

## Publisher's Note

All claims expressed in this article are solely those of the authors and do not necessarily represent those of their affiliated organizations, or those of the publisher, the editors and the reviewers. Any product that may be evaluated in this article, or claim that may be made by its manufacturer, is not guaranteed or endorsed by the publisher.

## References

[B1] Arampatzis A. Karamanidis K. Morey-Klapsing G. De Monte G. Stafilidis S. (2007). Mechanical properties of the triceps surae tendon and aponeurosis in relation to intensity of sport activity. J. Biomech. 40, 1946–1952. 10.1016/j.jbiomech.2006.09.005 17101142

[B2] Behara B. Jacobson B. H. (2017). Acute effects of deep tissue foam rolling and dynamic stretching on muscular strength, power, and flexibility in division i linemen. J. Strength Cond. Res. 31, 888–892. 10.1519/JSC.0000000000001051 26121431

[B3] Behm D. G. Alizadeh S. Anvar S. H. Drury B. Granacher U. Moran J. (2021). Non-local acute passive stretching effects on range of motion in healthy adults: a systematic review with meta-analysis. Sport. Med. 51, 945–959. 10.1007/s40279-020-01422-5 33459990

[B4] Behm D. G. Blazevich A. J. Kay A. D. McHugh M. (2016). Acute effects of muscle stretching on physical performance, range of motion, and injury incidence in healthy active individuals: a systematic review. Appl. Physiol. Nutr. Metab. 41, 1–11. 10.1139/apnm-2015-0235 26642915

[B5] Behm D. G. Chaouachi A. (2011). A review of the acute effects of static and dynamic stretching on performance. Eur. J. Appl. Physiol. 111, 2633–2651. 10.1007/s00421-011-1879-2 21373870

[B6] Behm D. G. Wilke J. (2019). Do self-myofascial release devices release myofascia? Rolling mechanisms: a narrative review. Sport. Med. 49, 1173–1181. 10.1007/s40279-019-01149-y 31256353

[B7] Borenstein M. Hedges L. V. Higgins J. P. T. Rothstein H. R. (2009). Introduction to Meta-Analysis. Available online at: www.wiley.com. (accessed: February 2, 2021). 10.1002/9780470743386

[B8] Bradbury-Squires D. J. Noftall J. C. Sullivan K. M. Behm D. G. Power K. E. Button D. C. (2015). Roller-massager application to the quadriceps and knee-joint range of motion and neuromuscular efficiency during a lunge. J. Athl. Train. 50, 133–140. 10.4085/1062-6050-49.5.03 25415414 PMC4495431

[B9] Cheatham S. W. Kolber M. J. Cain M. Lee M. (2015). The effects of self-myofascial release using a foam roll or roller massager on joint range of motion, muscle recovery, and performance: a systematic review. Int. J. Sports Phys. Ther. 10, 827–838. 26618062 PMC4637917

[B10] Connolly G. Hammer R. L. Powell J. A. O'connor P. L. (2020). A single bout of foam rolling increases flexibility of the hip adductor muscles without compromising strength. Int. J. Exerc. Sci. 13, 938–949. 32922650 10.70252/ATYJ6383 PMC7449344

[B11] Donti O. Panidis I. Terzis G. Bogdanis G. (2019). Gastrocnemius medialis architectural properties at rest and during stretching in female athletes with different flexibility training background. Sports 7:39. 10.3390/sports7020039 30781768 PMC6410170

[B12] Fairall R. R. Cabell L. Boergers R. J. Battaglia F. (2017). Acute effects of self-myofascial release and stretching in overhead athletes with GIRD. J. Bodyw. Mov. Ther. 21, 648–652. 10.1016/j.jbmt.2017.04.001 28750979

[B13] Fallon J. B. Macefield V. G. (2007). Vibration sensitivity of human muscle spindles and golgi tendon organs. Muscle Nerve 36, 21–29. 10.1002/mus.20796 17471568

[B14] Folli A. Ghirlanda F. Cescon C. Schneebeli A. Weber C. Vetterli P. . (2021). A single session with a roller massager improves hamstring flexibility in healthy athletes: a randomized placebo-controlled crossover study. Sport Sci. Health 17, 717–724. 10.1007/s11332-021-00737-8

[B15] Germann D. El Bouse A. Shnier J. Abdelkader N. Kazemi M. (2018). Effects of local vibration therapy on various performance parameters: a narrative literature review. J. Can. Chiropr. Assoc. 62, 170–181. Available at: /pmc/articles/PMC6319432/?report=abstract (accessed August 6, 2020). 30662072 PMC6319432

[B16] Giovanelli N. Vaccari F. Floreani M. Rejc E. Copetti J. Garra M. . (2018). Short-term effects of rolling massage on energy cost of running and power of the lower limbs. Int. J. Sports Physiol. Perform. 13, 1337–1343. 10.1123/ijspp.2018-0142 29745784

[B17] Halperin I. Aboodarda S. J. Button D. C. Andersen L. L. Behm D. G. (2014). Roller massager improves range of motion of plantar flexor muscles without subsequent decreases in force parameters. Int. J. Sport. Phys. Ther. 9, 92–102. 24567860 PMC3924613

[B18] Higgins J. P. T. Thompson S. G. Deeks J. J. Altman D. G. (2003). Measuring inconsistency in meta-analyses. Br. Med. J. 327, 557–560. 10.1136/bmj.327.7414.557 12958120 PMC192859

[B19] Hopkins W. G. Marshall S. W. Batterham A. M. Hanin J. (2009). Progressive statistics for studies in sports medicine and exercise science. Med. Sci. Sport. Exerc. 41, 3–13. 10.1249/MSS.0b013e31818cb278 19092709

[B20] Janot J. Malin B. Cook R. Hagenbucher J. Draeger A. Jordan M. . (2013). Effects of self myofascial release and static stretching on anaerobic power output. J. Fit. Res. 2, 41–54.

[B21] Kaneda H. Takahira N. Tsuda K. Tozaki K. Kudo S. Takahashi Y. . (2020). Effects of tissue flossing and dynamic stretching on hamstring muscles function. J. Sport. Sci. Med. 19, 681–689. 33239941 PMC7675630

[B22] Kato E. Kanehisa H. Fukunaga T. Kawakami Y. (2010). Changes in ankle joint stiffness due to stretching: the role of tendon elongation of the gastrocnemius muscle. Eur. J. Sport Sci. 10, 111–119. 10.1080/17461390903307834

[B23] Kay A. D. Blazevich A. J. (2012). Effect of acute static stretch on maximal muscle performance: a systematic review. Med. Sci. Sport. Exerc 44, 154–164. 10.1249/MSS.0b013e318225cb27 21659901

[B24] Kay A. D. Husbands-Beasley J. Blazevich A. J. (2015). Effects of contract-relax, static stretching, and isometric contractions on muscle-tendon mechanics. Med. Sci. Sports Exerc. 47, 2181–2190. 10.1249/MSS.0000000000000632 25668401

[B25] Konrad A. Bernsteiner D. Budini F. Reiner M. M. Glashüttner C. Berger C. . (2020). Tissue flossing of the thigh increases isometric strength acutely but has no effects on flexibility or jump height. Eur. J. Sport Sci. 10.1080/17461391.2020.1853818. [Epub ahead of print]. 33315544

[B26] Konrad A. Budini F. Tilp M. (2017a). Acute effects of constant torque and constant angle stretching on the muscle and tendon tissue properties. Eur. J. Appl. Physiol. 117, 1649–1656. 10.1007/s00421-017-3654-5 28624851 PMC5506206

[B27] Konrad A. Močnik R. Nakamura M. Sudi K. Tilp M. (2021a). The impact of a single stretching session on running performance and running economy: a scoping review. Front. Physiol. 11:630282. 10.3389/fphys.2020.630282 33551850 PMC7857312

[B28] Konrad A. Močnik R. Titze S. Nakamura M. Tilp M. (2021b). The influence of stretching the hip flexor muscles on performance parameters. a systematic review with meta-analysis. Int. J. Environ. Res. Public Health 18:1936. 10.3390/ijerph18041936 33671271 PMC7922112

[B29] Konrad A. Reiner M. M. Thaller S. Tilp M. (2019). The time course of muscle-tendon properties and function responses of a five-minute static stretching exercise. Eur. J. Sport Sci. 19, 1195–1203. 10.1080/17461391.2019.1580319 30821657 PMC6816483

[B30] Konrad A. Stafilidis S. Tilp M. (2017b). Effects of acute static, ballistic, and PNF stretching exercise on the muscle and tendon tissue properties. Scand. J. Med. Sci. Sport. 27, 1070–1080. 10.1111/sms.12725 27367916 PMC5479471

[B31] Konrad A. Tilp M. (2018). Muscle and tendon tissue properties of competitive soccer goalkeepers and midfielders: a pilot study. Ger. J. Exerc. Sport Res. 48, 245–251. 10.1007/s12662-018-0510-7

[B32] Konrad A. Tilp M. (2020a). The acute time course of muscle and tendon tissue changes following one minute of static stretching. Curr. Issues Sport Sci. 5:3. 10.15203/CISS_2020.003

[B33] Konrad A. Tilp M. (2020b). The time course of Muscle-Tendon unit function and structure following three minutes of static stretching. J. Sport. Sci. Med. 19, 52–58. 32132827 PMC7039016

[B34] Kopec T. J. Bishop P. A. Esco M. R. (2017). Influence of dynamic stretching and foam rolling on vertical jump. Athl. Train. Sport. Heal. Care 9, 33–38. 10.3928/19425864-20161003-01

[B35] Kubo K. Kanehisa H. Kawakami Y. Fukunaga T. (2001). Influence of static stretching on viscoelastic properties of human tendon structures *in vivo*. J. Appl. Physiol. 90, 520–527. 10.1152/jappl.2001.90.2.520 11160050

[B36] Lee C. L. Chu I. H. Lyu B. J. Chang W. D. Chang N. J. (2018). Comparison of vibration rolling, nonvibration rolling, and static stretching as a warm-up exercise on flexibility, joint proprioception, muscle strength, and balance in young adults. J. Sports Sci. 36, 2575–2582. 10.1080/02640414.2018.1469848 29697023

[B37] Lopez-Samanes A. Del Coso J. Hernández-Davó J. L. Moreno-Pérez D. Romero-Rodriguez D. Madruga-Parera M. . (2021). Acute effects of dynamic versus foam rolling warm-up strategies on physical performance in elite tennis players. Biol. Sport. 38, 595–601. 10.5114/biolsport.2021.101604 PMC8670807 34937969

[B38] Lyu B. J. Lee C. L. Chang W. D. Chang N. J. (2020). Effects of vibration rolling with and without dynamic muscle contraction on ankle range of motion, proprioception, muscle strength and agility in young adults: a crossover study. Int. J. Environ. Res. Public Health 17:354. 10.3390/ijerph17010354 31948000 PMC6982037

[B39] Maher C. G. Sherrington C. Herbert R. D. Moseley A. M. Elkins M. (2003). Reliability of the PEDro scale for rating quality of randomized controlled trials. Phys. Ther. 83, 713–721. 10.1093/ptj/83.8.713 12882612

[B40] Mayer I. Hoppe M. W. Freiwald J. Heiss R. Engelhardt M. Grim C. . (2019). Different effects of foam rolling on passive tissue stiffness in experienced and nonexperienced athletes. J. Sport Rehabil. 29, 926–933. 10.1123/jsr.2019-0172 31775121

[B41] Mizuno T. Umemura Y. (2016). Dynamic stretching does not change the stiffness of the muscle-tendon unit. Int. J. Sports Med. 37, 1044–1050. 10.1055/s-0042-108807 27676152

[B42] Moher D. Liberati A. Tetzlaff J. Altman D. G. (2009). Preferred reporting items for systematic reviews and meta-analyses: the PRISMA statement. PLoS Med. 6:e1000097. 10.1371/journal.pmed.1000097 19621072 PMC2707599

[B43] Monte A. Zignoli A. (2021). Muscle and tendon stiffness and belly gearing positively correlate with rate of torque development during explosive fixed end contractions. J. Biomech. 114:110110. 10.1016/j.jbiomech.2020.110110 33302182

[B44] Morales-Artacho A. J. Lacourpaille L. Guilhem G. (2017). Effects of warm-up on hamstring muscles stiffness: cycling vs foam rolling. Scand. J. Med. Sci. Sport. 27, 1959–1969. 10.1111/sms.12832 28124382

[B45] Moran J. Ramirez-Campillo R. Liew B. Chaabene H. Behm D. G. García-Hermoso A. . (2021). Effects of bilateral and unilateral resistance training on horizontally orientated movement performance: a systematic review and meta-analysis. Sport. Med. 51, 225–242. 10.1007/s40279-020-01367-9 33104995

[B46] Nakamura M. Onuma R. Kiyono R. Yasaka K. Sato S. Yahata K. . (2021). Acute and prolonged effects of different durations of foam rolling on range of motion, muscle stiffness, and muscle strength. J. Sport. Sci. Med. 20, 62–68. 10.52082/jssm.2021.62 33707988 PMC7919347

[B47] Pişirici P. Ekiz M. B. Ilhan C. (2020). Investigation of the acute effect of myofascial release techniques and dynamic stretch on vertical jump performance in recreationally active individuals. J. Phys. Educ. Sport 20, 1569–1579. 10.7752/jpes.2020.03215

[B48] Reid J. C. Greene R. Young J. D. Hodgson D. D. Blazevich A. J. Behm D. G. (2018). The effects of different durations of static stretching within a comprehensive warm-up on voluntary and evoked contractile properties. Eur. J. Appl. Physiol. 118, 1427–1445. 10.1007/s00421-018-3874-3 29721606

[B49] Reiner M. M. Glashüttner C. Bernsteiner D. Tilp M. Guilhem G. Morales-Artacho A. . (2021). A comparison of foam rolling and vibration foam rolling on the quadriceps muscle function and mechanical properties. Eur. J. Appl. Physiol. 121, 1461–1471. 10.1007/s00421-021-04619-2 33638016 PMC8064982

[B50] Sagiroglu I. Kurt C. Pekünlü E. Özsu I. (2017). Residual effects of static stretching and self-myofascial-release exercises on flexibility and lower body explosive strength in well-trained combat athletes. Isokinet. Exerc. Sci. 25, 135–141. 10.3233/IES-160656

[B51] Samson M. Button D. C. Chaouachi A. Behm D. G. (2012). Effects of dynamic and static stretching within general and activity specific warm-up protocols. J. Sports Sci. Med. 11, 279–285. 24149201 PMC3737866

[B52] Smith J. C. Pridgeon B. Hall M. C. (2018). Acute effect of foam rolling and dynamic stretching on flexibility and jump height. J. Strength Cond. Res. 32, 2209–2215. 10.1519/JSC.0000000000002321 29621115

[B53] Somers K. Aune D. Horten A. Kim J. Rogers J. (2020). Acute effects of gastrocnemius/soleus self-myofascial release versus dynamic stretching on closed-chain dorsiflexion. J. Sport Rehabil. 29, 287–293. 10.1123/jsr.2018-0199 30747565

[B54] Su H. Chang N. J. Wu W. L. Guo L. Y. Chu I. H. (2017). Acute effects of foam rolling, static stretching, and dynamic stretching during warm-ups on muscular flexibility and strength in young adults. J. Sport Rehabil. 26, 469–477. 10.1123/jsr.2016-0102 27736289

[B55] Trajano G. Seitz L. Nosaka K. Blazevich A. (2019). Passive muscle stretching impairs rapid force production and neuromuscular function in human plantar flexors. Eur. J. Appl. Physiol. 119, 2673–2684. 10.1007/s00421-019-04244-0 31650306

[B56] Wiewelhove T. Döweling A. Schneider C. Hottenrott L. Meyer T. Kellmann M. . (2019). A meta-analysis of the effects of foam rolling on performance and recovery. Front. Physiol. 10:376. 10.3389/fphys.2019.00376 31024339 PMC6465761

[B57] Wilke J. Müller A. L. Giesche F. Power G. Ahmedi H. Behm D. G. (2020). Acute effects of foam rolling on range of motion in healthy adults: a systematic review with multilevel meta-analysis. Sport. Med. 50, 387–402. 10.1007/s40279-019-01205-7 31628662

